# Molecular Mobility and Stability Studies of Amorphous Imatinib Mesylate

**DOI:** 10.3390/pharmaceutics11070304

**Published:** 2019-07-01

**Authors:** Bożena Karolewicz, Agata Górniak, Dominik M. Marciniak, Igor Mucha

**Affiliations:** 1 Department of Drug Form Technology, Wroclaw Medical University, Borowska 211 A, 50-556 Wroclaw, Poland; 2 Laboratory of Elemental Analysis and Structural Research, Wroclaw Medical University, Borowska 211 A, 50-556 Wroclaw, Poland; 3 Department of Analytical Chemistry, Wroclaw Medical University, Borowska 211 A, 50-556 Wroclaw, Poland

**Keywords:** imatinib mesylate, amorphous form, stability, molecular mobility, thermal analysis, mean relaxation-time constant

## Abstract

The proposed study examined the characterization and stability of solid-state amorphous imatinib mesylate (IM) after 15 months under controlled relative humidity (60 ± 5%) and temperature (25 ± 2 °C) conditions. After 2 weeks, and 1, 3, 6, and 15 months, the samples were characterized using differential scanning calorimetry (DSC), thermogravimetric analysis (TGA), X-ray powder diffractometry (XRPD), attenuated total reflectance-Fourier transform infrared spectroscopy (ATR-FTIR) and scanning electron microscopy (SEM). Additionally, the amorphous form of imatinib mesylate was obtained via supercooling of the melt in a DSC apparatus, and aged at various temperatures (3, 15, 25 and 30 °C) and time periods (1–16 h). Glass transition and enthalpy relaxation were used to calculate molecular-relaxation-time parameters. The Kohlrausch–Williams–Watts (KWW) equation was applied to fit the experimental enthalpy-relaxation data. The mean molecular-relaxation-time constant (*τ*) increased with decreasing ageing temperature. The results showed a high stability of amorphous imatinib mesylate adequate to enable its use in solid dosage form.

## 1. Introduction

**Figure 1:**
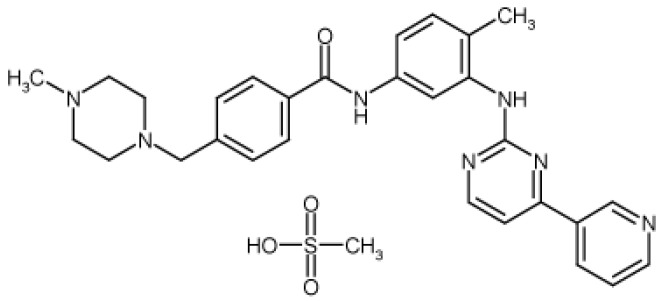
Chemical structure of imatinib mesylate prepared by means of ChemDraw Professional software (PerkinElmer, Waltham, MA, USA).

Amorphous substances form a separate class of solids, which are distinct from the more common and well-known crystalline solid. Crystalline materials consist of a three-dimensional long-range structure, which is not present in the amorphous state. Furthermore, compared to crystalline state, the positioning of amorphous state molecules are more irregular, as well as possessing high internal energy and specific volume, which improves dissolution and bioavailability [1,2,3]. The most common methods of amorphization include rapid precipitation from solution, quench-cooling, spray-drying, freeze-drying, and hot melt extrusion [4,5,6,7,8]. Mechanical stress associated with grinding and milling can also transform crystalline solids into amorphous form [9]. Amorphous substances are generally hygroscopic, with high solubility and bioavailability, and can possess superior compression characteristics than corresponding crystals [10,11]. The role of molecular mobility in time-dependent nucleation, chemical degradation, crystallization, and structural collapse of amorphous materials has been recognized in several studies [12,13,14]. In terms of stability, the amorphous state can unexpectedly transform into the crystalline state during production or storage [15,16]. Information regarding physicochemical stability of the amorphous state of drugs is invaluable in accurately determining pharmaceutical dosage [17,18]. To classify amorphous substances as stable, they must exhibit relaxation times comparable to their shelf life (3 years for pharmaceuticals) [19]. Therefore, the objective of this study is to characterize the behavior and performance of imatinib mesylate (IM) in its amorphous state in order to predict its stability as a pharmaceutical raw material. IM (Figure 1), i.e., 4-(4-methyl-piperazin-1-ylmethyl)-*N*-u[4-methyl-3-(4-pyridin-3-yl)pyrimidine-2-ylamino)phenyl]benzamide methanesulfonate, is a tyrosine-kinase inhibitor commonly used in the treatment of chronic myeloid leukemia [20,21,22] and gastrointestinal stromal tumors [23,24,25]. It can be useful in the treatment of atherosclerosis [26,27], thrombosis [28,29], and restenosis [30,31]. Imatinib therapy is a good option for unresectable, recurrent, or metastatic dermatofibrosarcoma protuberans [32,33,34,35,36,37]. IM is known to exist in various polymorphic forms, including hydrated and solvated forms, as well as hydrated and anhydrous amorphous forms [38,39,40,41,42,43,44]. The most extensively examined IM polymorphs are *α*- and *β*-form. Grillo et al. showed that these forms transform into amorphous IM upon grinding, followed by conversion to the crystalline phase during aging or thermal treatment. Furthermore, recent studies have found that α and *β*-forms are enantiotropically related. Detailed reports have confirmed these solid forms based on crystallographic and spectroscopic techniques [45,46].

To the best of our knowledge, information related to the stability and characterization of IM amorphous form in the literature is non-excitant. However, there is a strong relationship between molecular mobility and stability of amorphous form in pharmaceutical substances. To predict the life expectancy of pure amorphous compounds in the study, we searched for correlations between crystallization tendency of glass formers with molecular mobility and thermodynamic properties of the amorphous state. It was recognized that storage well below *T*_g_ prevents crystallization and ensures a physically stable drug during its shelf-life [13,47,48]. In this paper, the physical stability of IM was investigated using thermal methods by considering the onset temperature and enthalpy relaxation at the glass transition of the ageing time. The stability of IM in the amorphous state was estimated by the mean molecular relaxation-time constant “*τ*” and relaxation-time distribution parameter *β* using the Kohlrausch–Williams–Watts (KWW) equation. Moreover, the physicochemical characteristics of IM at time ageing were determined by thermal techniques X-ray powder diffractometry (XRPD), attenuated total reflectance-Fourier transform infrared spectroscopy (ATR-FTIR) and scanning electron microscopy (SEM). This is the first time such a study of amorphous IM was presented.

## 2. Materials and Methods

### 2.1. Materials

Crystalline imatinib mesylate *α* form used in this study was synthetized in a new continuous-flow microwave reactor, described in [49] and donated by Silesian Catalysts Sp. z o.o (Wrocław, Poland). Residual solvents (below 0.5%) were determined using gas chromatography–mass spectrometry (GC-MS) method. Reference material: commercial IM of a grade useful for R&D was purchased from LC Laboratories (Woburn, MA, USA). According to the certificate attached by the manufacturer, the declared purity of the substance was 94.66% (by nuclear magnetic resonance (NMR) method). The verification of purity by the method of differential scanning calorimetry (DSC) in our study confirmed 93.99% ± 0.08 purity.

Our previous studies confirmed that both commercial and synthetized IM occur in crystalline *α* form [50].

### 2.2. Preparation of Amorphous Sample

Preparation of the amorphous form of a drug for physical stability studies was achieved by melting the drug in a stainless-steel beaker on a hot plate, followed by subsequent quenching by cooling the melt over crushed ice (method A). The quench-cooled product was ground, sieved using 315 μm sieve, and examined after 2 weeks, 1 month, 3 months, 6 months, and 15 months, respectively.

Additionally, the amorphous form of IM was obtained by supercooling the melt in a DSC apparatus (method B), and aged at various temperatures (3, 15, 25, and 30 °C) for periods of time between 1 and 16 h.

### 2.3. Physical Stability Studies

The physical stability of amorphous IM was monitored for fifteen months under controlled relative humidity (60 ± 5%) and temperature (25 ± 2 °C), which was the long-term stability condition, selected based on Guideline for Industry Q1A (R2) Stability Testing of new Drug Substances and Products [51]. Periodically (0 day, 2 weeks, 1 month, 3 months, 6 months, and 15 months), the samples were removed and examined for solid-state transitions using DSC, TGA, XRD, ATR-FTIR, and SEM methods.

### 2.4. Differential Scanning Calorimetry (DSC)

Differential scanning calorimeter DSC 214 Polyma instrument (Netzsch, Selb, Germany) equipped with an Intracooler IC70 (Netzsch, Selb, Germany) was used. Measurements of the amorphous samples were performed using the heat-flow measurement method at a heating rate of 10 °C per minute over a temperature range of 10–250 °C in a nitrogen dynamic atmosphere (25 mL/min). The samples of approx. 8.5 mg were placed in an aluminium pan (25 µL) with a pierced lid, and subsequently sealed. An empty pan of the same type was employed as reference. DSC peak area and transition temperatures were determined using Netzsch Proteus^®^ 7.1.0 analysis software (Netzsch, Selb, Germany). The apparatus was calibrated using standard samples from calibration set 6.239.2-91.3 supplied by Netzsch (Selb, Germany). All samples were weighed on a Sartorius CPA225D-0CE analytical balance (Sartorius AG, Gottingen, Germany) with a resolution of 0.01 mg.

**Figure 2:**
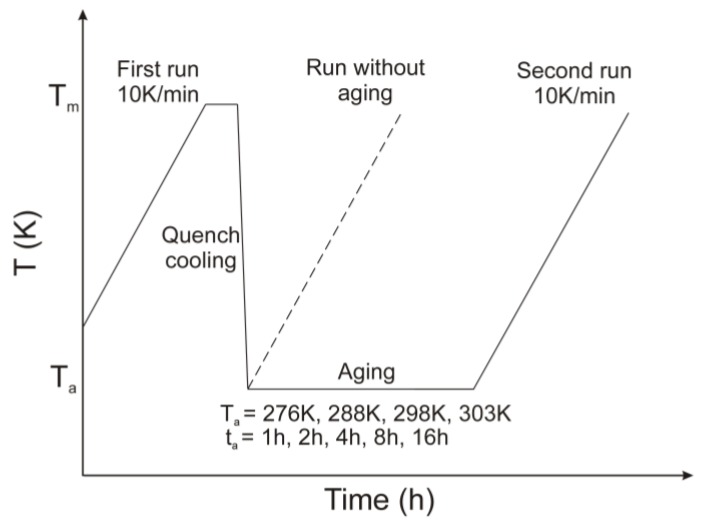
Temperature programme applied in differential scanning calorimetry (DSC) during the ageing experiments.

In this study, the ageing experiments of IM crystals were also performed. The schematic representation of the entire temperature program is illustrated in Figure 2. The samples were melted in an aluminium pan at 175 °C and maintained for 5 min to avoid incomplete melting. The melt was quench-cooled at approx. 200 °C per minute cooling rate to different ageing temperatures (*T_a_*) (3, 15, 25, and 30 °C) for a predetermined period of ageing time (*t_a_*) (1, 2, 4, 8, and 16 h). Following the specified aging time, the glassy materials were heated at 10 °C per minute to 185 °C. A cycle for unaged sample was processed. This second heating determined the change heat capacity (*ΔC_p_*), the extrapolated onset glass transition temperature (*T_g_*) and the enthalpy recovery (*ΔH_t_*).

### 2.5. Thermogravimetric Analysis (TGA)

The thermal stability of the samples was investigated by thermogravimetric analysis using TG 209 F1 Libra (Netzsch, Selb, Germany) thermobalance. The experiments were carried out in alumina crucibles (150 μL), under nitrogen atmosphere (25 mL/min) at a heating rate of 10 °C per minute. TGA/DTG curves were obtained in the temperature range of 25–600 °C. Netzsch Proteus^®^ 7.1.0 analysis software (Selb, Germany) was used to determine the mass change of the samples and DTG curves.

### 2.6. Powder X-ray Diffraction Analysis (XRPD)

Crystalline/amorphous nature of IM was determined using Bruker D2 PHASER diffractometer (BRUKER AXS, Karlsruhe, Germany) with a LynxEYe detector. Cu Kα radiation (1.5418 Å) was used at 30 kV and 10 mA. Diffractograms were obtained in the Bragg-Brentano (*θ*/2*θ*) horizontal geometry between 7° and 40° (2*θ*) (step size 0.02° and 0.1 s/step) at 295 K. XRD patterns were analyzed using the software Diffrac.EVA v.2.1 (BRUKER AXS, Karlsruhe, Germany)

### 2.7. Attenuated Total Reflectance-Fourier Transform Infrared Spectroscopy (ATR-FTIR)

A Nicolet 380 FTIR (Thermo Scientific, Waltham, MA, USA) analyzer equipped with OMNIC analysis software was used for FTIR spectra registration. Spectra were collected in attenuated total reflectance (ATR) mode. A small amount of each sample was placed on the diamond crystal and scanned over a wavelength of 400 cm^−1^ to 4000 cm^−1^ with a resolution of 4 cm^−1^.

### 2.8. Scanning Electron Microscopy (SEM)

Scanning electron microscope (SEM) images were taken on Zeiss EVO MA25 (Carl Zeiss NTS GmbH, Oberkochen, Germany) apparatus at an accelerating voltage of 20 kV. The samples were covered with gold and palladium (60:40; sputter current, 40 mA; sputter time, 50 s) using a Quorum machine (Quorum International, Fort Worth, TX, USA).

## 3. Results and Discussion

**Figure 3:**
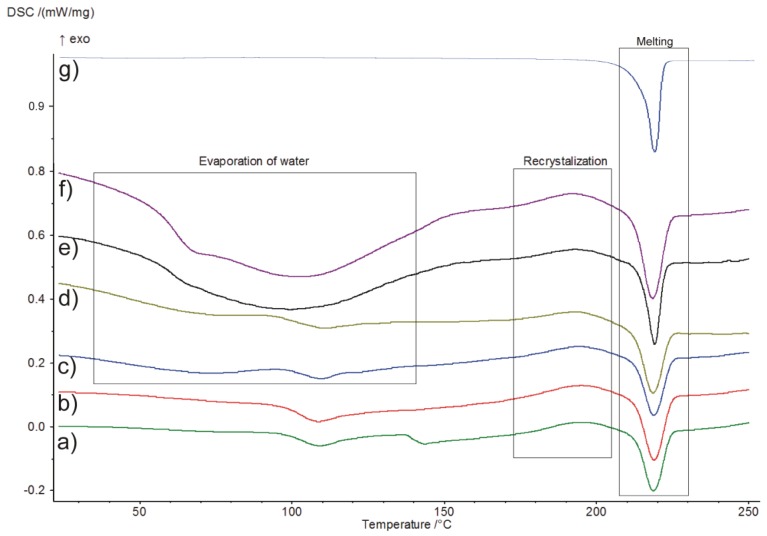
Differential scanning calorimetry (DSC) curves of amorphous imatinib mesylate (IM) stored at 25 °C under controlled relative humidity at different ageing times: (**a**) day 0, (**b**) 2 weeks, (**c**) 1 month, (**d**) 3 months, (**e**) 6 months, and (**f**) 15 months, and (**g**) crystalline IM.

DSC curves of amorphous IM samples stored at 25 °C under controlled relative humidity at different ageing times are presented in Figure 3. After 1 month (Figure 3c–f), it can be observed that the moisture is adsorbed by samples, which also corroborated with the results obtained from TGA. Water evaporation occurred between 40–130 °C, followed by recrystallization and melting of the sample. The glass transition temperature of amorphous IM with increasing ageing time at an ageing temperature of 25 °C was determined at approx. 100 °C. No significant changes were observed at the ageing temperature. In the case of samples stored longer than 6 and 15 months (Figure 3e,f), the glass transition was difficult to detect due to moisture absorption. Compared to DSC curves of IM amorphous form, only one thermal (endothermic) effect corresponding to the melting was visible for crystalline form (Figure 3g). DSC parameters of amorphous imatinib mesylate are presented in Table 1.

**Figure 4:**
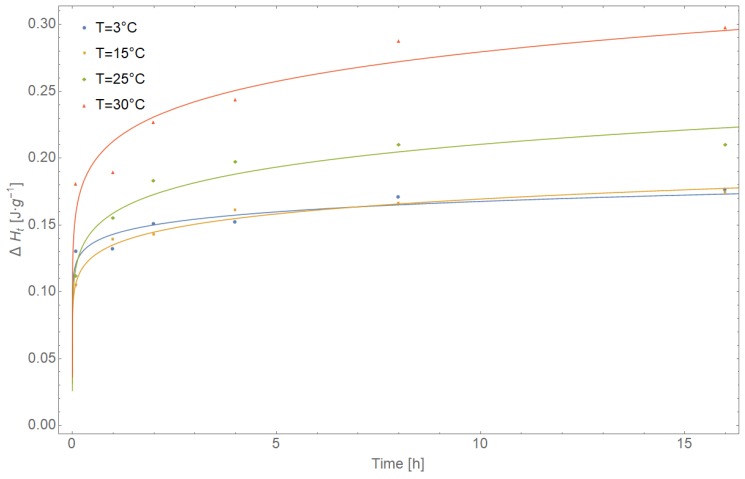
*ΔH_t_* as a function of ageing time for various ageing temperatures.

Based on DSC curves recorded at various temperatures (3, 15, 25, and 30 °C), enthalpy recovery (*ΔH_t_*) data for amorphous IM were obtained. Using the KWW equation, the average relaxation time constant *τ* and dimensionless relaxation time distribution parameter *β* were estimated (Table 2). Figure 4 shows the best fits of the KWW equation to the experimental data (solid lines). The initial parameters were *τ* = 100 s and *β* = 0.5. The KWW equation is as follows:

(1) $$\emptyset_{t}=1-\frac{\Delta H_{t}}{\Delta H_{\infty }}=\mathrm{Exp}\left[-{\left(\frac{t}{\tau }\right)}^{\beta }\right],$$

where ∅_t_ is the extent of impending relaxation at the annealing temperature, *∆H_∞_* is the maximum enthalpy recovery, *t* is the ageing time*, τ* is the mean relaxation-time constant, and *β* is the non-exponential parameter, respectively.

*∆H_∞_* was calculated from the following equation:

(2) $$\Delta H_{\infty }=\Delta C_{p}\cdot \left(T_{g}-T_{a}\right),$$

where *∆C_p_* is the heat capacity change at *T_g_* and *T_a_* ageing temperature.

Values of *τ* and *β* estimators presented in Table 3 were determined using two non-linear optimization algorithms: Levenberg–Marquardt and Quasi–Newton, which minimize the loss function L according to the equation:

(3) $$L\left(\tau ,\beta \right)=\min RRS\left(\tau ,\beta \right)=\min\sum {\left(y_{i}-f\left(x_{i},\hat{\tau },\hat{\beta }\right)\right)}^{2},$$

where RSS is the residual sum of squares, $y_{i}$ is the experimental values, and $f\left(x_{i}\right)$ the values predicted by the estimated model.

The statistical significance of *τ* and *β* estimators was evaluated using the t-test, assuming the confidence level *p* = 0.05, and 95% confidence interval (−95% CI/+95% CI). The quality of matching the KWW functions to the experimental data was determined by coefficients of determination *R*^2^ (*R*-squared). The correctness of the entire model was additionally checked by residual analysis (Table 3). For each measurement point, the value predicted by the model $f\left(x_{i},\hat{\tau },\hat{\beta }\right)$ was determined as $L\left(\tau ,\beta \right)=\sum {\left(y_{i}-f\left(x_{i},\hat{\tau },\hat{\beta }\right)\right)}^{2}$. The values of residues were determined according to the formula $L_{i}=\left(y_{i}-f\left(x_{i},\hat{\tau },\hat{\beta }\right)\right)$. A standard error, and 95% confidence interval for $f\left(x_{i},\hat{\tau },\hat{\beta }\right)$ and values of the entire model were also determined. The normality of the distribution of calculated $L_{i}$ residues was assessed using the Shapiro–Wilk W-test with the assumed confidence level p = 0.05. The distribution of standardized values $L_{i}$ is shown in Figure 4. The times *t_50%_* listed in Table 3 are defined as the times required for half completion (50%) of the possible maximum enthalpy relaxation at a single relaxation temperature, which corresponds to the value of relaxation time τ in the KWW equation when *∅_t_* = 0.50. By comparing *τΦ*(*t*) = 50% values of each glass at each aging temperature, it was found that as aging temperature decreased, *τΦ*(*t*) = 50% values increased dramatically, hence, the rate of the aging process dramatically decreased at lower aging temperatures. A rapid decrease in *t_50%_* values was observed with increasing aging temperatures from 4.91695 × 10^33^ h at 3 °C to 8.21992 × 10^16^ h at 30 °C, which again slowed the aging process at lower temperatures. Calculated mean molecular relaxation time constants for amorphous IM range from 3.609324 × 10^39^ to 6.393168 × 10^21^ s, which are comparable with values obtained for griseofulvin, tolbutamide, troglitazone, and simvastatin [52].

The width of relaxation-time distribution parameter *β* ranging from 0 to 1 were characterized. The smaller the *β* value, a greater distribution of molecular motion deviates from a single exponential behavior was obtained. Estimated values of *β* parameters were comparable at different ageing temperatures but vary significantly from the unit. Therefore, this indicates a distribution of time scale rather than one single relaxation time. Furthermore, with an increase in ageing temperature the mean relaxation-time constants *τ* decreased.

The statistical analysis presented in Table 3 was constructed using the following programs: Mathematica ver. 10.0 by Wolfram Research and Statistica^®^ ver. 13.1 from Dell.

**Figure 5:**
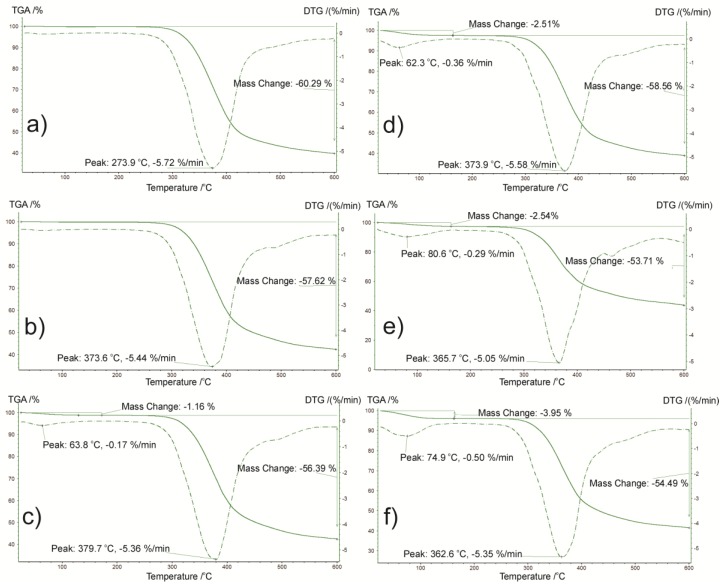
Thermogravimetric analysis (TGA)/derivative thermogravimetry (DTG) curves of amorphous imatinib mesylate (IM) at ageing temperature 25 °C at different ageing times: (**a**) day 0, (**b**) 2 weeks, (**c**) 1 month, (**d**) 3 months, (**e**) 6 months, and (**f**) 15 months.

Thermogravimetry and derivative thermogravimetry (DTG) was used to identify the thermal stability of amorphous IM. TGA and DTG curve of the substance at 0 time and after a specified time are presented in Figure 5. The thermal decomposition parameters are as follows: temperature of maximum weight loss rate (*T_m_*); extrapolated onset temperature of decomposition (*T_Onset_*); rate of mass loss, which corresponds to *T_m_*; extrapolated temperature at which the degradation process ends (*T_Endset_*); temperature at which 0.5 wt.% and 1.0 wt.% loss occurs and the amount of residue (Res) at 600 °C, are presented in Table 4.

**Figure 6:**
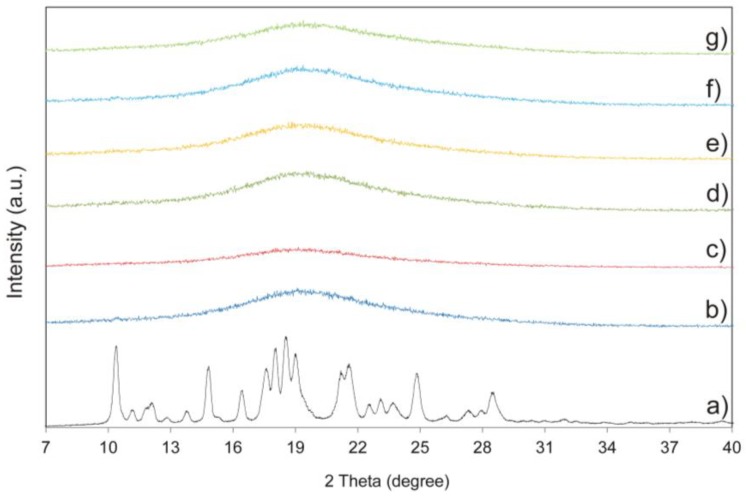
X-ray powder diffractometry (XRPD) pattern of crystalline imatinib mesylate (IM) (**a**) and amorphous IM stored at 25 °C under controlled relative humidity at different ageing times: (**b**) day 0, (**c**) 2 weeks, (**d**) 1 month, (**e**) 3 months, (**f**) 6 months, and (**g**) 15 months.

From the above results it can be extrapolated that the thermal degradation of the analyzed samples occurs in one step. The average temperature of maximum weight loss rate for the main effect was 371.57 °C, which corresponds to 5.42% min^−1^, the rate of mass loss. As shown in Figure 6c-f, additional mass changes in the temperature range 25–140 °C was observed, possibly due to a loss of loosely bound water. The presence of water is evident from the DCS curves (Figure 3c–f). Moisture content fluctuates depending on ageing times from 1.16 % (1 month aging) to 3.95 % (15 months aging). A fall in the baseline above 270 °C was due to substance degradation. The average temperature at which 0.5 wt.% loss occurs was observed at approximately 274 °C, while the average extrapolated onset temperature of decomposition was 325 °C. The differentiation at c.a. 50 °C between *T_Onset_* and T_0.5 wt.%_ should be taken into consideration during thermal stability assessment.

According to TGA/DTG curve registered for the crystalline form of the substance, decomposition occurs in one step. The thermal decomposition parameters: *T_m_* = 376.0 °C, rate mass loss = 5.61 %·min^−1^, *T_Onset_* = 325.6 °C, *T_Endset_* = 424.1 °C, *T_0.5 wt.%_* = 278.6 °C, *T_1.0 wt.%_* = 291.7 °C, and *Res* = 40.29% were determined. In the temperatures ranging between 25–140 °C no mass loss was observed related to moisture.

XRPD pattern of crystalline IM confirms its crystalline nature (Figure 6a). The studied sample displayed peaks at 2*θ* values of 10.38°, 11.17°, 11.89°, 12.11°, 12.85°, 13.78°, 14.82°, 16.42°, 17.62°, 18.02°, 18.53°, 19.03°, 21.19°, 19.75°, 21.19°, 21.55°, 22.56°, 23.12°, 23.66°, 24.83°, 26.24°, 27.33°, 27.93°, 28.46°, and 31.92. The obtained results correspond to *α* form and literature data [50]. The amorphous sample of the drug stored at 25 °C under controlled relative humidity at different ageing times is shown in Figure 6b–g. All diffractograms display a broad amorphous halo with the absence of sharp diffraction peaks signals characteristic for crystalline form.

**Figure 7:**
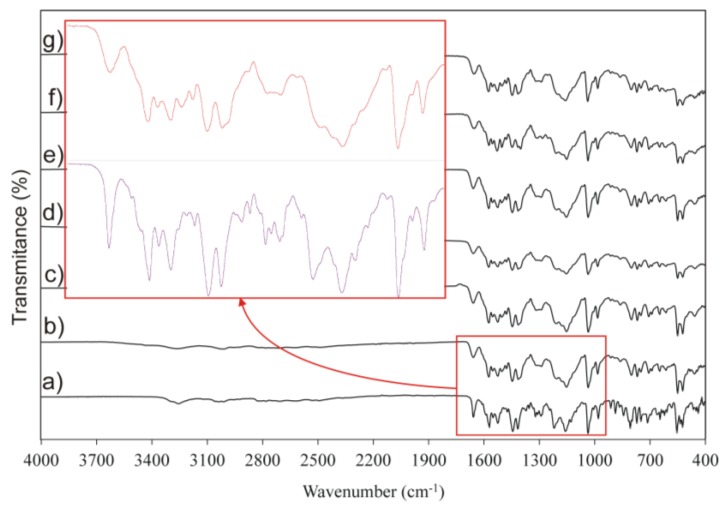
Attenuated total reflectance-Fourier transform infrared spectroscopy (ATR-FTIR) spectra crystalline imatinib mesylate (IM) (**a**) and amorphous IM stored at 25 °C under controlled relative humidity at different ageing times: (**b**) day 0, (**c**) 2 weeks, (**d**) 1 month, (**e**) 3 months, (**f**) 6 months, and (**g**) 15 months.

ATR-FTIR spectra of IM samples in the 4000–400 cm^−1^ region are presented in Figure 7. The obtained results correspond well with literature data [53]. ATR-FTIR showed characteristic bands at 3256 cm^−1^ (N–H stretching vibration), 1657 cm^−1^ (C=O band), 1570, 1525, and 1444 cm^−1^ (aromatic C=C, C=N stretching vibration), 1158 cm^−1^ (C–N stretching vibration), 1036 cm^−1^ (C–O stretching vibration), and 807 cm^−1^ (aromatic C–H deformations out of plane). ATR-FTIR spectra for crystalline and amorphous IM samples were qualitatively similar. The spectra of amorphous forms showed peak broadening attributed to the relative disorder of the molecules in the amorphous form, resulting in a broader distribution of bond lengths and energies with respect their crystalline counterparts.

**Figure 8:**
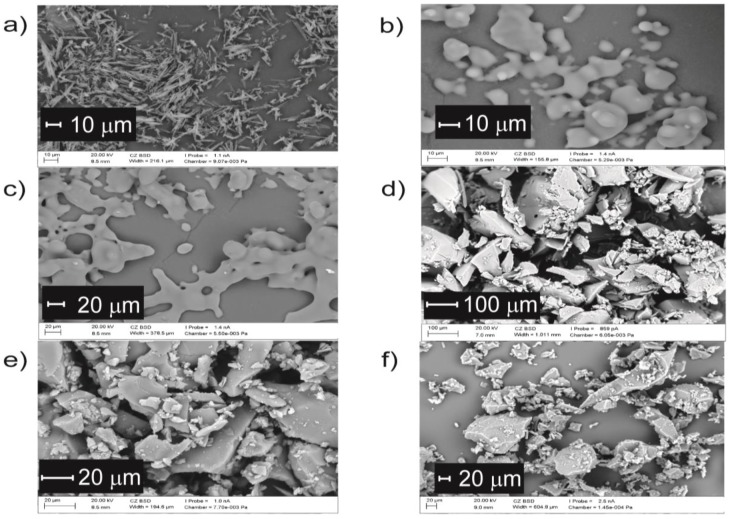
Imatinib mesylate (IM) microscopic images (**a**) crystalline form (magnification 1390x); amorphous form after (**b**) 2 weeks (magnification 1930x), (**c**) 1 month (magnification 794x), (**d**) 3 months (magnification 297x), (**e**) 6 months (magnification 1540x), and (**f**) 15 months of storage (magnification 500x).

In order to estimate the morphology of IM samples, scanning electron microscopy (SEM) analysis was performed. SEM images of crystalline and amorphous IM are shown in Figure 8. Synthetized IM exists as a needles-shape crystalline structure (Figure 8a). IM particles, observed on SEM images taken after 2 weeks (Figure 8b) and 1 month (Figure 8c) of storage, reveal an irregular spherical shape, characteristic for amorphous state. After 3, 6, and 15 months of storage (Figure 8d–f, respectively) the shape of IM particles become less compact, but remain irregular and do not resemble the needles.

## 4. Conclusions

A delicate balance exists among the chemical and physical properties of amorphous materials. Slight alterations or disturbances can have a major influence on stability, as well as future production and dissolution characteristics of the final product [54]. Unfortunately, the storage of such materials can promote unwanted transformation to their more stable crystalline form [55].

Herein, we showed efficient methodology for the preparation of amorphous IM via its crystalline form. The process eliminated unwanted chemical degradation and the presence of amorphous IM was confirmed using analytical techniques: DSC, ATR-FTIR, and XRPD. Glass transition and enthalpy relaxation were experimentally determined and used to calculate molecular relaxation-time parameters. The KWW equation fitted the experimental enthalpy relaxation data well. Two non-linear optimization algorithms: Levenberg–Marquardt and Quasi–Newton were applied to predict the long-term amorphous stability of IM. It was determined that the mean molecular relaxation time constant (τ) increased with decreasing ageing temperature.

By calculating the relaxation time of amorphous IM, it was possible to estimate the timescale of undesirable solid-state degradation or crystallization as a function of temperature. The calculated mean molecular-relaxation time constants for amorphous IM confirmed the long-term stability of the examined samples. This amorphous substance could be a potential candidate for the development of novel drug products.

## Figures and Tables

**Table 1:** Differential scanning calorimetry (DSC) parameters of amorphous imatinib mesylate (IM).

Time	*T_p_* [^o^C]	*T_Onset_* [^o^C]	*T_Endset_* [^o^C]	*ΔH_f_* [J g^−1^]	*T_g_* [°C]	*ΔC_p_* [J g^−1^]
0 day	218.7	212.4	224.5	10.73	100.9	0.168
2 weeks	218.9	212.3	224.5	11.42	101.0	0.238
1 month	218.8	212.1	224.8	10.52	99.0	0.140
3 months	218.5	212.0	224.4	11.37	100.0	0.208
6 months	219.1	213.8	222.6	10.70	105.2	0.301
15 months	218.3	211.9	224.3	11.76	106.4	0.302

*T_p_* - peak melting temperature; *T_Onset_* - onset melting temperature; *T_Endset_* - endset melting temperature; *ΔH_f_ -* enthalpy of fusion; *T_g_*_-_ glass transition; *ΔC_p_* - heat capacity.

**Table 2:** The results for the best fit to the Kohlrausch–Williams–Watts (KWW) equation.

*T* [*°C*]	Maximum Enthalpy *∆H_∞_* [*J∙g^−1^*]	Estimated Parameters								*R*-Value	*t_50%_* [*h*]
		Mean molecular Relaxation Time Constant *τ* [*s*]				Relaxation Time Distribution Parameter *β*					
		Estimate *τ*	Standard Error *SE*	Confidence Interval *−95% CI/+95% CI*	*P*-Value	Estimate *β*	Standard Error *SE*	Confidence Interval *−95% CI/+95% CI*	*P*-Value		
3	43.4	3.6 × 10^39^	2.8 × 10^35^	2.80 × 10^35^/ 1.72 × 10^36^	0.0161	0.0689	0.000267	0.0682/ 0.0697	1.29 × 10^−9^	0.998	4.92 × 10^33^
15	38.3	2.95 ×∙10^28^	8.62 × 10^23^	5.99 × 10^24^/ 1.04 × 10^25^	0.000216	0.098	0.000206	0.0979/ 0.099	1.15 × 10^−10^	0.999	1.98 × 10^23^
25	34.1	4.13 ×∙10^22^	2.04 × 10^18^	6.23 × 10^18^/ 1.67 × 10^19^	0.00245	0.122	0.000570	0.121/ 0.124	2.84 × 10^−9^	0.997	5.73 × 10^17^
30	32.0	6.39 ×∙10^21^	3.93 × 10^17^	7.66 × 10^17^/ 2.79 × 10^18^	0.006289	0.119	0.000724	0.117/ 0.121	8.17 × 10^−9^	0.997	8.22 × 10^16^

**Table 3:** The results of the Residual Analysis.

T [°C]	Time [h]	Observed Value $y_{i}$	Predicted Value $f\left(x_{i},\hat{\tau },\hat{\beta }\right)$	Residual $L_{i}=\left(y_{i}-f\left(x_{i},\hat{\tau },\hat{\beta }\right)\right)$	Standard Error $SD_{i}$	Confidence Interval		Residual Sum of Squares RRS	Shapiro Wilk W/p
						*−95% CI_i_*	*+95% CI_i_*		
3 °C	0.1	0.130	0.122	0.00791	0.0027	0.115	0.130	0.0002599	*W* = 0.9426/ *p* = 0.6808
	1.0	0.132	0.143	−0.01145	0.0031	0.134	0.152		
	2.0	0.151	0.150	0.00056	0.0033	0.141	0.159		
	4.0	0.152	0.157	−0.00527	0.0034	0.148	0.167		
	8.0	0.170	0.165	0.00535	0.0035	0.155	0.175		
	16.0	0.176	0.173	0.00309	0.0037	0.163	0.183		
15 °C	0.1	0.105	0.108	−0.00315	0.0013	0.104	0.111	0.0000712	*W* = 0.8655/ *p* = 0.20908
	1.0	0.139	0.135	0.00349	0.0016	0.131	0.140		
	2.0	0.142	0.145	−0.00254	0.0017	0.140	0.149		
	4.0	0.160	0.155	0.00557	0.0018	0.150	0.160		
	8.0	0.166	0.166	−0.00014	0.0018	0.161	0.171		
	16.0	0.174	0.177	−0.00341	0.0020	0.172	0.183		
25 °C	0.1	0.112	0.120	−0.00821	0.0032	0.111	0.129	0.0004607	*W* = 0.9089/ *p* = 0.42970
	1.0	0.155	0.159	−0.00383	0.0040	0.148	0.170		
	2.0	0.183	0.173	0.0101	0.0042	0.161	0.185		
	4.0	0.197	0.188	0.00890	0.0046	0.176	0.201		
	8.0	0.210	0.205	0.00531	0.0049	0.191	0.218		
	16.0	0.210	0.223	−0.0131	0.0052	0.208	0.237		
30 °C	0.1	0.181	0.162	0.0188	0.0052	0.147	0.176	0.0012054	*W* = 0.9593/ *p* = 0.81450
	1.0	0.189	0.212	−0.0234	0.0065	0.195	0.231		
	2.0	0.227	0.231	−0.00406	0.0069	0.212	0.250		
	4.0	0.243	0.251	−0.00738	0.0074	0.230	0.271		
	8.0	0.287	0.272	0.0151	0.0078	0.250	0.294		
	16.0	0.298	0.296	0.00206	0.0084	0.272	0.319		

**Table 4:** The thermal decomposition parameters of amorphous imatinib mesylate (IM).

Time	*T_m_* [^o^C]	Rate of Mass Loss [% min^−1^]	*T_Onset_* [^o^C]	*T_Endset_* [^o^C]	*Res* [%]	*T_0.5 wt.%_* [^o^C]	*T_1.0 wt.%_* [^o^C]	Moisture Content [%]
0 day	373.9	5.72	326.2	419.6	39.71	271.5	287.6	-
2 weeks	373.6	5.44	327.7	419.4	42.37	269.3	287.2	-
1 month	379.7	5.36	327.8	419.3	42.48	275.5	291.5	1.16
3 months	373.9	5.58	326.3	419.8	38.92	275.7	289.5	2.51
6 months	365.7	5.05	322.6	411.9	43.75	274.6	280.3	2.54
15 months	362.6	5.35	319.9	410.4	41.55	278.4	288.1	3.95

*T_Onset_*—extrapolated onset temperature of decomposition; *T_m_*—temperature of maximum weight loss rate; *T_Endset_*—extrapolated temperature at which the degradation process ends; *T_0.5 wt.%_*—temperature at which 0.5 wt.% loss occurs; *T_1.0 wt.%_*—temperature at which 1.0 wt.% loss occurs; *Res—*amount of residue at 600 °C.

## References

[1] Yu L. (2001). Amorphous pharmaceutical solids: Preparation, characterization and stabilization. Adv. Drug Deliv. Rev..

[2] Hancock B.C., Parks M. (2000). What is the true solubility advantage for amorphous pharmaceuticals?. Pharm. Res..

[3] Nielsen L.H., Gordon S., Holm R., Selen A., Rades T., Mullertz A. (2013). Preparation of an amorphous sodium furosemide salt improves solubility and dissolution rate and leads to a faster T-max after oral dosing to rats. Eur. J. Pharm. Biopharm..

[4] Craig D.Q.M., Royall P.G., Kett V.L., Hopton M.L. (1999). The relevance of the amorphous state to pharmaceutical dosage forms: Glassy drugs and freeze dried systems. Int. J. Pharm..

[5] Alvarez-Nunez F.A., Leonard M.R. (2004). Formulation of a poorly soluble drug using hot melt extrusion: The amorphous state as an alternative. Am. Pharm. Rev..

[6] Kim J.-S., Kim M.-S., Park H.J., Jin S.-J., Lee S., Hwang S.-J. (2008). Physicochemical properties and oral bioavailability of amorphous atorvastatin hemi-calcium using spray-drying and SAS process. Int. J. Pharm..

[7] Aucamp M., Liebenberg W., Strydom S.J., van Tonder E.C., de Villiers M.M. (2012). Physicochemical properties of amorphous roxithromycin prepared by quench cooling of the melt or desolvation of a chloroform solvate. AAPS PharmSciTech..

[8] Einfalt T., Planinšek O., Hrovat K. (2013). Methods of amorphization and investigation of the amorphous state. Acta Pharm..

[9] Karmwar P., Graeser K., Gordon K.C., Strachan C.J., Rades T. (2012). Effect of different preparation methods on the dissolution behaviour of amorphous indomethacin. Eur. J. Pharm. Biopharm..

[10] Vranić E. (2004). Amorphous Pharmaceutical Solids. Bosn. J. Basic Med. Sci..

[11] Hancock B.C., Carlson G.T., Ladipo D.D., Langdon B.A., Mullarney M.P. (2002). Comparison of the mechanical properties of the crystalline and amorphous forms of a drug substance. Int. J. Pharm..

[12] Zhou D., Zhang G.G., Law D., Grant D.J.W., Schmitt E.A. (2002). Physical stability of amorphous pharmaceuticals: Importance of configurational thermodynamic quantities and molecular mobility. J. Pharm. Sci..

[13] Yoshioka S., Aso Y. (2007). Correlations between molecular mobility and chemical stability during storage of amorphous pharmaceuticals. J. Pharm. Sci..

[14] Zhou D., Zhang G.G., Law D., Grant D.J.W., Schmitt E.A. (2008). Thermodynamics, molecular mobility and crystallization kinetics of amorphous griseofulvin. Mol. Pharm..

[15] Shete G., Puri V., Kumar L., Bansal A.K. (2010). Solid state characterization of commercial crystalline and amorphous atorvastatin calcium samples. AAPS PharmSciTech..

[16] Grčman M., Vrečer F., Meden A. (2002). Some physico-chemical properties of doxazosin mesylate polymorphic forms and its amorphous state. J. Therm. Anal. Calorim..

[17] Bellur A.E., Karlığa B. (2015). Quantitative determination of two polymorphic forms of imatinib mesylate in a drug substance and tablet formulation by X-ray powder diffraction, differential scanning calorimetry and attenuated total reflectance Fourier transform infrared spectroscopy. J. Pharm. Biomed. Anal..

[18] Martena V., Censi R., Hoti E., Malaj L., Di Martino P. (2012). Physicochemical characterization of nicergoline and cabergoline in its amorphous state. J. Therm. Anal. Calorim..

[19] Chawla G., Bansal A.K. (2009). Molecular Mobility and Physical Stability of Amorphous Irbesartan. Sci Pharm..

[20] Druker B.J., Talpaz M., Resta D.J., Peng B., Buchdunger E., Ford J.M., Lydon N.B., Kantarjian H., Capdeville R., Ohno-Jones S. (2001). Efficacy and safety of a specific inhibitor of the BCR-ABL tyrosine kinase in chronic myeloid leukemia. N. Engl. J. Med..

[21] Cortes J.E., Baccarani M., Guilhot F., Druker B.J., Branford S., Kim D.W., Pane F., Pasquini R., Goldberg S.L., Kalaycio M. (2010). Phase III, randomized, open-label study of daily imatinib mesylate 400 mg versus 800 mg in patients with newly diagnosed, previously untreated chronic myeloid leukemia in chronic phase using molecular end points: Tyrosine kinase inhibitor optimization and selectivity study. J. Clin. Oncol..

[22] Miyamura K., Ohnishi K., Ohtake S., Usui N., Nakaseko C., Fujita H., Fujisawa S., Sakura T., Okumura H., Iriyama N. (2019). Randomized study of imatinib for chronic myeloid leukemia: Comparing standard dose escalation with aggressive escalation. Blood Adv..

[23] Tolomeo M., Dieli F., Gebbia N., Simoni D. (2009). Tyrosine kinase inhibitors for the treatment of chronic myeloid leukemia. Anticancer Agents Med. Chem..

[24] Demetri G.D., von Mehren M., Blanke C.D., Van den Abbeele A.D., Eisenberg B., Roberts P.J., Heinrich M.C., Tuveson D.A., Singer S., Janicek M. (2002). Efficacy and safety of imatinib mesylate in advanced gastrointestinal stromal tumors. N. Engl. J. Med..

[25] Ben Ami E., Demetri G.D. (2016). A safety evaluation of imatinib mesylate in the treatment of gastrointestinal stromal tumor. Expert Opin. Drug Saf..

[26] Lassila M., Allen T.J., Cao Z., Thallas V., Jandeleit-Dahm K.A., Candido R.M., Cooper E. (2004). Imatinib attenuates diabetes-associated atherosclerosis. Arterioscler. Thromb. Vasc. Biol..

[27] Masuda S., Nakano K., Funakoshi K., Zhao G., Meng W., Kimura S., Matoba T., Miyagawa M., Iwata E., Sunagawa K. (2011). Imatinib mesylate-incorporated nanoparticle-eluting stent attenuates in-stent neointimal formation in porcine coronary arteries. J. Atheroscler. Thromb..

[28] Ojeda-Uribe M., Merieau S., Guillon M., Aujoulat O., Hinschberger O., Eisenmann J.C., Kenizou D., Debliquis A., Veyradier A., Chantrel F. (2016). Secondary thrombotic microangiopathy in two patients with Philadelphia-positive hematological malignancies treated with imatinib mesylate. J. Oncol. Pharm. Pract..

[29] Fukada I., Araki K., Kobayashi K., Shibayama T., Hatano M., Takahashi S., Iwase T., Ohno S., Ito Y. (2016). Imatinib could be a new strategy for pulmonary hypertension caused by pulmonary tumor thrombotic microangiopathy in metastatic breast cancer. Springer Plus..

[30] Zohlnhofer D., Hausleiter J., Kastrati A., Mehilli J., Goos C., Schuhlen H., Pache J., Pogatsa-Murray G., Heemann U., Dirschinger J. (2005). A randomized, double-blind, placebo-controlled trial on restenosis prevention by the receptor tyrosine kinase inhibitor imatinib. J. Am. Coll. Cardiol..

[31] Park Y.J., Min S.K., Min S.I., Kim S.J., Ha J. (2015). Effect of imatinib mesylate and rapamycin on the preformed intimal hyperplasia in rat carotid injury model. Ann.Surg. Treat. Res..

[32] Lemm D., Muegge L.O., Hoeffken K., Aklan T., Mentzel T., Thorwarth M., Schultze-Mosgau S. (2008). Remission with Imatinib mesylate treatment in a patient with initially unresectable dermatofibrosarcoma protuberans—A case report. Oral. Maxillofac. Surg..

[33] Rutkowski P., Van Glabbeke M., Rankin C.J., Ruka W., Rubin B.P., Debiec-Rychter M., Lazar A., Gelderblom H., Sciot R., Lopez-Terrada D. (2010). Imatinib mesylate in advanced dermatofibrosarcoma protuberans: Pooled analysis of two phase II clinical trials. J. Clin. Oncol..

[34] Stacchiotti S., Pedeutour F., Negri T., Conca E., Marrari A., Palassini E., Collini P., Keslair F., Morosi C., Gronchi A. (2011). Dermatofibrosarcoma protuberans-derived fibrosarcoma: Clinical history, biological profile and sensitivity to imatinib. Int. J. Cancer..

[35] Wicherts D.A., Coevorden F., Klomp H.M., Huizum M.A., Kerst J.M., Haas R.L.M., van Boven H.H., van der Hage J.A. (2013). Complete resection of recurrent and initially unresectable dermatofibrosarcoma protuberans downsized by Imatinib. World J. Surg. Oncol..

[36] Wang C., Luo Z., Chen J., Zheng B., Zhang R., Chen Y., Shi Y. (2015). Target therapy of unresectable or metastatic dermatofibrosarcoma protuberans with imatinib mesylate: An analysis on 22 chinese patients. Medicine.

[37] Tatai T., Gomi D., Fukushima T., Kobayashi T., Sekiguchi N., Sakamoto A., Sasaki S., Koizumi T., Sano K. (2016). Effectiveness of Imatinib Mesylate Treatment in a Patient with Dermatofibrosarcoma Protuberans with Pulmonary and Pancreatic Metastases. Intern. Med..

[38] Khunt M.D., Patil N.S., Pagire H.S., Pradhan N.S., Valgeirsson J. (2008). Anhydrous amorphous imatinib mesylate. U.S. Patent.

[39] Zimmermann J., Sutter B., Buerger H. (2002). Crystal modification of a N-phenyl-2-pyrimidineamine derivative, processes for its manufacture and its use. U.S. Patent.

[40] Bandi P.R., Kura R.R., Rapolu R.R., Dasari M.R., Kesireddy S.C.R. (2004). Novel polymorphs of imatinib mesylate.

[41] Patel H.V., Jani R.J., Thennati R. (2006). Imatinib mesylate crystal form and process for preparation thereof.

[42] Haas P.D., Koc F., Karliga B., Atici E.B., Sivasligil R. (2011). Polymorphs of imatinib. Eur. Patent.

[43] Pathi S.L., Puppala R., Kankan R.N., Rao D.R. (2012). Stable crystal form of imatinib mesylate and process for the preparation thereof. U.S. Patent.

[44] Lin M., Wu Y., Rohani S. (2019). A kinetic study of crystallization process of imatinib mesylate with polymorphic transformation phenomenon. J. Cryst. Growth..

[45] Grillo D., Polla G., Vega D. (2012). Conformational polymorphism on imatinib mesylate: Grinding effects. J. Pharm. Sci..

[46] Srivastava A., Joshi B.D., Tandon P., Ayala A.P., Bansal A.K., Grillo D. (2013). Study of polymorphism in imatinib mesylate: A quantum chemical approach using electronic and vibrational spectra. Spectrochim. Acta Part A Mol. Biomol. Spectrosc..

[47] Bhardwajand S.P., Suryanarayanan R. (2012). Molecular mobility as an effective predictor of the physical stability of amorphous trehalose. Mol. Pharmaceutics.

[48] Schammé B., Couvrat N., Malpeli P., Delbreilh L., Dupray V., Dargent É., Coquerel G. (2015). Crystallization kinetics and molecular mobility of an amorphous active pharmaceutical ingredient: A case study with Biclotymol. Int. J. Pharm..

[49] Reszke E., Łojkowski W., Łapczyński M.R., Niklewicz P.G. (2011). Microwave reactorfor chemical reactions. PL Patent.

[50] Mucha I., Baranowski P., Owczarek A., Gajda M., Pluta J., Górniak A., Niklewicz P., Karolewicz B. (2016). Thermal stability and decompositions kinetics under non-isothermalconditions of imatinib mesylate α form. J. Pharm. Biom. Anal..

[51] (2003). Guideline for Industry Q1A (R2) Stability Testing of New Drug Substances and Products, International Conference on Harmonisation. https://www.fda.gov/media/71707/download.

[52] Graeser K.A., Patterson J.E., Zeitler J.A., Gordon K.C., Rades T. (2009). Correlating thermodynamic and kinetic parameters with amorphous stability. Eur. J. Pharm. Sci..

[53] Veverka M., Šimon P., Lokaj J., Veverková E. (2012). Crystal habit modifications of imatinib mesylate under various precipitation condition. Monatsh. Chem..

[54] Chadha R., Kashid N., Jain D.V.S. (2005). Characterization and quantification of amorphous content in same selected parenteral cephalosporins by calorimetric method. J. Therm. Anal. Calorim..

[55] Alhalaweh A., Alzghoul A., Mahlin D., Bergström C.A.S. (2015). Physical stability of drugs after storage above and below the glass transition temperature: Relationship to glass-forming ability. Int. J. Pharm..

